# Ornstein–Uhlenbeck Adaptation as a Mechanism for Learning in Brains and Machines

**DOI:** 10.3390/e26121125

**Published:** 2024-12-22

**Authors:** Jesús García Fernández, Nasir Ahmad, Marcel van Gerven

**Affiliations:** Department of Machine Learning and Neural Computing, Donders Institute for Brain, Cognition and Behaviour, Radboud University, 6500HB Nijmegen, The Netherlands; jesus.garciafernandez@donders.ru.nl (J.G.F.); n.ahmad@donders.ru.nl (N.A.)

**Keywords:** Ornstein–Uhlenbeck process, neuromorphic computing, continuous-time neural networks, reward prediction error, stochastic neurotransmitter release

## Abstract

Learning is a fundamental property of intelligent systems, observed across biological organisms and engineered systems. While modern intelligent systems typically rely on gradient descent for learning, the need for exact gradients and complex information flow makes its implementation in biological and neuromorphic systems challenging. This has motivated the exploration of alternative learning mechanisms that can operate locally and do not rely on exact gradients. In this work, we introduce a novel approach that leverages noise in the parameters of the system and global reinforcement signals. Using an Ornstein–Uhlenbeck process with adaptive dynamics, our method balances exploration and exploitation during learning, driven by deviations from error predictions, akin to reward prediction error. Operating in continuous time, Ornstein–Uhlenbeck adaptation (OUA) is proposed as a general mechanism for learning in dynamic, time-evolving environments. We validate our approach across a range of different tasks, including supervised learning and reinforcement learning in feedforward and recurrent systems. Additionally, we demonstrate that it can perform meta-learning, adjusting hyper-parameters autonomously. Our results indicate that OUA provides a promising alternative to traditional gradient-based methods, with potential applications in neuromorphic computing. It also hints at a possible mechanism for noise-driven learning in the brain, where stochastic neurotransmitter release may guide synaptic adjustments.

## 1. Introduction

One of the main properties of any intelligent system is that it has the capacity to learn. This holds for biological systems, ranging from bacteria and fungi to plants and animals [1,2,3,4], as well as for engineered systems designed by artificial intelligence (AI) researchers [5,6,7]. Modern intelligent systems, such as those used in machine learning, typically rely on gradient descent for learning by minimizing error gradients [8,9,10]. While gradient-based methods have driven significant advances in AI [6], their reliance on exact gradients, centralized updates, and complex information pathways limits their applicability in biological and neuromorphic systems.

In contrast, biological learning likely relies on different mechanisms, as organisms often lack the exact gradient information and centralized control that gradient descent requires [11,12]. Neuromorphic computing, inspired by these principles, aims to replicate the distributed, energy-efficient learning of biological systems [13,14]. However, integrating traditional gradient-based methods into neuromorphic hardware has proven challenging, highlighting a critical gap: the need for gradient-free learning mechanisms that exclusively rely on operations that are local in space and time [15,16].

To address this, alternative learning principles to gradient descent have been proposed for both rate-based [17,18,19,20,21] and spike-based models [22,23,24,25]. A class of methods that leverages inherent noise present in biological systems to facilitate learning is perturbation-based methods [26,27,28], which adjust the system’s parameters based on noise effects and global reinforcement signals, offering gradient-free, local learning suitable for biological or neuromorphic systems. Specifically, these methods inject random fluctuations (noise) into the system and evaluate the impact of this noise on performance through a reinforcement signal. Then, they adjust the system’s parameters to increase or decrease alignment with the output of the system featuring noise, depending on the feedback provided by the reinforcement signal. Within this framework, node perturbation methods [28,29,30,31,32,33,34] inject noise into the nodes, while weight perturbation methods [35,36,37,38,39] inject noise into the parameters. Particularly, node perturbation has been shown to approximate gradient descent updates on average [28], but it relies on a more stochastic exploration of the loss landscape rather than consistently following the steepest descent direction, as in gradient-based methods. Nevertheless, both approaches require generating two outputs per input—one noisy and one noise-free—to accurately measure the impact of the noise on the system, and access to the noise process, making them impractical in many real-world or biological scenarios. Reward-modulated Hebbian learning (RMHL) [40,41], a bio-inspired alternative, overcomes some of these limitations as it does not need a noiseless system nor access to the noise process to learn. Yet, RMHL often struggles to solve practical tasks efficiently, limiting their applicability.

In this work, we propose Ornstein–Uhlenbeck Adaptation (OUA), a novel learning mechanism that extends the strengths of perturbation-based methods while addressing some of their limitations. Like RMHL methods, it does not require a noiseless system or direct access to the noise process to learn. Differently, OUA introduces a mean-reverting Ornstein–Uhlenbeck (OU) process [42,43] to inject noise directly into system parameters, adapting their mean based on a global modulatory reinforcement signal. This signal is derived from deviations in error predictions, resembling a reward prediction error (RPE) thought to play a role in biological learning [44]. Unlike traditional methods, OUA operates in continuous time using differential equations, making it particularly suited for dynamic, time-evolving environments [45]. While RMHL was developed to model biological learning dynamics, OUA’s online nature and ability to adapt continually offer advantages in practical applications. Additionally, the simple and local nature of the proposed mechanism makes it well-suited for implementation on neuromorphic hardware.

We validate our approach across various experiments, including feedforward and recurrent systems, covering input–output mappings, control tasks, and a real-world weather forecasting task, all within a continuous-time framework. Additionally, we demonstrate that the method can be extended to a meta-learning setting by learning the system hyper-parameters. Finally, we discuss the implications of OUA, as our experiments demonstrate its efficiency and versatility, positioning it as a promising approach for both neuromorphic computing and understanding biological learning mechanisms.

## 2. Methods

### 2.1. Inference

Consider an inference problem, where the goal is to map inputs $x\left(t\right)\in R^{m}$ to outputs $y\left(t\right)\in R^{k}$ for $t\in R^{+}$. In a continual learning setting, this problem can be formulated as a stochastic state space model

(1) $$\begin{array}{cc} dz(t) & =f_{\theta }\left(z\left(t\right),x\left(t\right)\right)dt+d\zeta \left(t\right) \end{array}$$

(2) $$\begin{array}{cc} y(t) & =g_{\theta }\left(z\left(t\right),x\left(t\right)\right)+\epsilon \left(t\right) \end{array}$$

**Figure 1:**
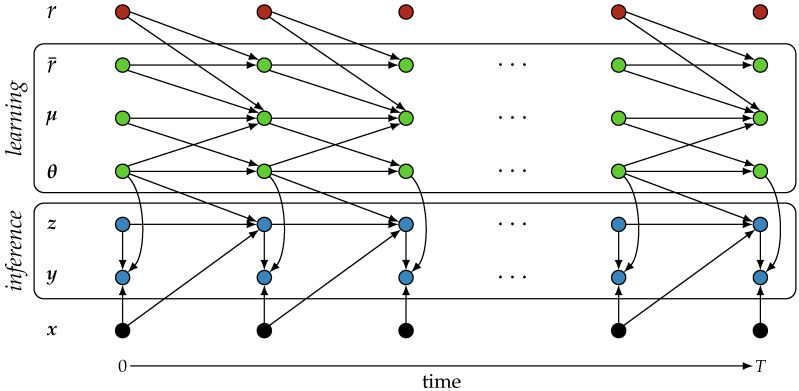
Dependency structure of the variables that together determine Ornstein–Uhlenbeck adaptation (hyper-parameters not shown). Variables $\bar{r}$, μ, and θ (green) are related to learning, whereas variables z and y (blue) are related to inference. The average reward estimate $\bar{r}$ depends on rewards *r* (red) that indirectly depend on the outputs y generated by the model. The output itself depends on input x (black).

Here, $\theta \in R^{n}$ are (learnable) parameters, $z\left(t\right)\in R^{d}$ is a latent process, $f_{\theta }\left(\cdot \right)$ and $g_{\theta }\left(\cdot \right)$ are nonlinear functions parameterized by θ, ζ(t) is process noise, and ϵ(t) is observation noise. We assume that we integrate the process from an initial time $t_{0}$ up to a time horizon *T*. We also refer to Equation (1) as a (latent) neural stochastic differential equation [46], where ‘neural’ refers to the use of learnable parameters θ. The dependency structure of the variables in OUA are depicted in Figure 1.

### 2.2. Reward Prediction

To enable learning, we assume the existence of a global scalar reward signal r(t), providing instantaneous feedback on the efficacy of the system’s output y(t). The goal is to adapt the parameters θ to maximize the cumulative reward

(3) $$G\left(t\right)=\int_{t_{0}}^{t}r\left(\tau \right)d\tau$$

as t→T. This can be expressed as an update equation G(t)=r(t)dt with initial state $G_{0}=G\left(t_{0}\right)=0$. We also refer to the expected cumulative reward as the return. To facilitate learning, the system maintains a moving average of the reward [47] according to

(4) $$d\bar{r}\left(t\right)=\rho \left(r\left(t\right)-\bar{r}\left(t\right)\right)dt.$$

This is equivalent to applying a low-pass filter to the reward with time-constant 1/ρ. We also refer to the difference $\delta_{r}\left(t\right)=r\left(t\right)-\bar{r}\left(t\right)$ as the reward prediction error, which can be interpreted as a global dopaminergic neuromodulatory signal, essential for learning in biological systems [44].

### 2.3. Learning

Often, learning is viewed as separate from inference. Here, in contrast, we cast learning and inference as processes that co-evolve over time by making the parameters part of the system dynamics. That is, in Equations (1) and (2), we assume that θ(t) evolves over time in parallel to the other variables. In this sense, the only distinction between learning and inference is that the former is assumed to evolve at a slower time scale compared to the latter.

The question remains of how to set up learning dynamics such that the parameters adapt towards a more desirable state. To this end, we define learning as a stochastic process evolving forward in time. Specifically, let us assume that parameter dynamics are given by an Ornstein–Uhlenbeck process

(5) dθ(t)=λ(μ(t)−θ(t))dt+ΣdW(t)

with μ(t) the mean parameter, λ the rate parameter, $\Sigma =\mathrm{diag}(\sigma_{1},\ldots ,\sigma_{n})$ the diffusion matrix, and $W\left(t\right)={\left(W_{1}\left(t\right),\ldots ,W_{n}\left(t\right)\right)}^{⊤}$ a stochastic process, which we take here to be a multivariate Wiener process. The Wiener process introduces stochastic perturbations, characterized by normally distributed increments with zero mean and variance proportional to d*t*, providing a mathematical model of random noise.

The OU process balances two key forces: (i) stability through mean-reversion via the term λ(μ(t)−θ(t)) and (ii) exploration through stochastic noise, as the term ΣdW(t) introduces randomness, allowing the system to explore the parameter space. Together, these terms embody the classical exploration–exploitation dilemma at the level of individual parameters. Exploration is driven by stochastic perturbations, which allow the parameters to sample a broad range of values. Exploitation, on the other hand, is guided by the mean-reverting force that nudges the parameters toward favorable regions identified by the current estimate of μ(t).

If we were to run Equation (5) in isolation, the parameters would simply fluctuate around the mean μ(t), with no directed learning. To enable adaptation, we define the mean parameter dynamics using an ordinary differential equation:

(6) $$d\mu (t)=\eta \delta_{r}\left(t\right)\left(\theta \left(t\right)-\mu \left(t\right)\right)dt$$

where η is the learning rate, and $\delta_{r}\left(t\right)$ is the RPE, acting as a global modulatory reinforcement signal. This reinforcement mechanism dynamically adjusts μ(t), shifting the focus of exploration towards regions associated with higher reward. The term (θ(t)−μ(t)) ensures that the adaptation of μ reflects the influence of recent stochastic updates to θ. The interplay between the stochastic term and the mean-reversion term results in probabilistic convergence behavior. As μ is refined through updates driven by $\delta_{r}$, the system converges to parameter values θ(T)∼N(μ(T),C) with mean μ(T) and stationary covariance $C=\Sigma \Sigma^{⊤}2\lambda$.

Since Equation (5) defines learning dynamics in terms of an Ornstein–Uhlenbeck process, we refer to our proposed learning mechanism as Ornstein–Uhlenbeck adaptation. OUA combines stochastic exploration with adaptive exploitation, making it particularly well-suited for continual learning in dynamic, time-evolving environments, such as those encountered in neuromorphic systems.

### 2.4. Experimental Validation

To test OUA as a learning mechanism, we designed experiments across several distinct scenarios. First, we analyzed the learning dynamics using a single-parameter model to gain insight into fundamental behavior. Subsequently, we examined recurrent and multi-parameter models to explore interactions among parameters and assess scalability. We then applied OUA to a real-world weather prediction task, forecasting temperature 24 h ahead based on current measurements of temperature, humidity, wind speed, wind direction (expressed as sine and cosine components), and atmospheric pressure. The dataset used in this task contains hourly recordings for Szeged, Hungary, collected between 2006 and 2016. Data can be obtained from https://www.kaggle.com/datasets/budincsevity/szeged-weather/ (accessed on the 20 December 2024). Outliers were removed using linear interpolation, and data were either standardized or whitened prior to further processing. To further assess OUA, we tackled a control problem known as the stochastic double integrator (SDI), where the objective was to maintain a particle’s position and velocity near zero despite the effects of Brownian motion. We refer to this control task as the stochastic double integrator (SDI) problem [48]. Finally, we extended our investigation to meta-learning, testing OUA’s ability to adapt hyper-parameters dynamically.

To implement OUA, we rely on numerical integration. The learning process is governed by a coupled system of stochastic and ordinary differential equations, defined in Equations (1), (2), and (4)–(6). Specifically, Equations (1) and (2) describe inference, while Equations (4)–(6) capture the learning dynamics. To integrate the system from the initial time $t_{0}$ to the time horizon *T*, we used an Euler–Heun solver implemented in the Python Diffrax package [49]. In each experiment, the step size for numerical integration was set to Δt=0.05. To interpolate inputs for the weather prediction task across the time window of interest, we used cubic Hermite splines with backward differences [50]. To ensure reproducibility, all scripts needed to replicate the results presented in this study are available via https://github.com/artcogsys/OUA (accessed on the 20 December 2024).

## 3. Results

In the following, we demonstrate OUA-based learning in increasingly complex systems. Here, we suppress the time index *t* from our notation to reduce clutter.

### 3.1. Learning a Single-Parameter Model

To analyze learning dynamics, we begin with a non-linear model containing a single learnable parameter θ. We assume that

(7) $$y=g_{\theta }\left(x\right)=\tanh\left(\theta x\right)$$

with input *x* and output *y*, and no latent state *z* is used. The input is given by a sinusoidal signal x(t)=sin(0.1t) and the reward is given by $r=-{\left(y-y^{*}\right)}^{2}$. The target output is given by $y^{*}=\tanh\left(\theta^{*}x\right)$, which is generated by a ground-truth parameter $\theta^{*}=1$. Thus, this setup focuses on supervised learning for a non-linear input–output mapping. The learning dynamics for the single-parameter model are described by the following stochastic differential equations:

(8) $$\begin{array}{cc} d\theta & =\lambda (\mu -\theta )dt+\sigma dW \end{array}$$

(9) $$\begin{array}{cc} d\mu & =\eta \delta_{r}\left(\theta -\mu \right)dt \end{array}$$

where $\bar{r}$ is the expected reward, $\delta_{r}=r-\bar{r}$ is the RPE and *W* is a standard Wiener process. Here, θ represents the model parameter and μ represents its mean.

**Figure 2:**
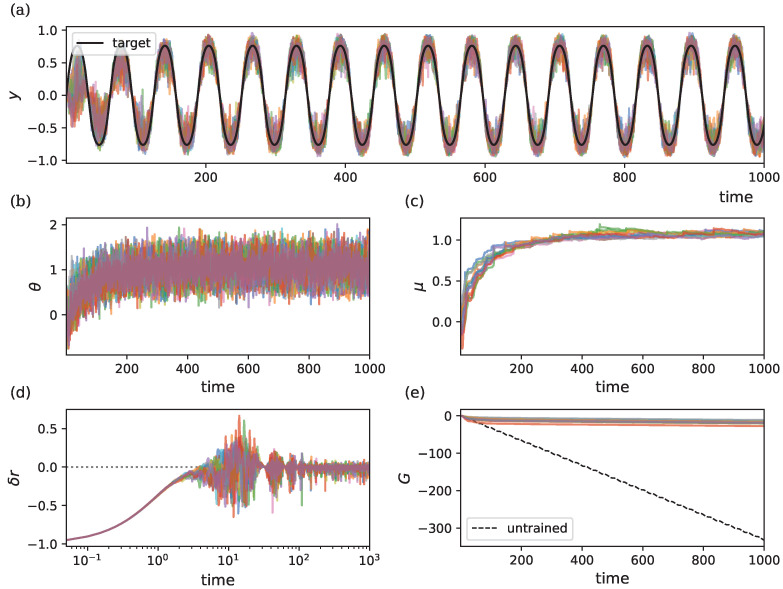
Dynamics of a single-parameter model across 15 random seeds (each color represents a different seed) with ρ=λ=η=1 and σ=0.3. Initial conditions are ${\bar{r}}_{0}=-1$, $\theta_{0}=0$ and $\mu_{0}=0$. The target output is generated by a ground-truth parameter $\theta^{*}=1$. (**a**) Target output vs. model output (**b**) Evolution of θ over time. (**c**) Evolution of μ over time. (**d**) RPE $\delta_{r}$ over time, shown on a logarithmic axis to better visualize initial convergence. The dotted line denotes zero reward prediction error. (**e**) Cumulative reward *G* over time, showing improvement with learning compared to the untrained model (dashed line).

Figure 2 illustrates the learning dynamics of this model, simulated over 15 trials using different random seeds. Figure 2a depicts the target output vs. the model output for the different random seeds. In Figure 2b,c, we observe the trajectories of θ and μ as they converge towards values that allow the model to approximate the target output. Due to the stochastic nature of the dynamics, convergence exhibits probabilistic behavior, leading to variability in θ (and thus μ) around their optimal values. Nonetheless, OUA ensures the model’s output closely follows the target, even in the presence of continuous noise.

Figure 2d shows how the RPE $\delta_{r}$ tends to zero over time. Despite achieving convergence, $\delta_{r}$ remains noisy due to the intrinsic stochasticity in the parameters. Figure 2e displays the cumulative reward *G* over time. The dashed line represents the cumulative reward for an untrained model ($\theta =\theta_{0}$). The results demonstrate that learning significantly improves accumulated reward, with variability in the cumulative reward arising from differences in the time it takes for θ to converge in individual trials.

**Figure 3:**
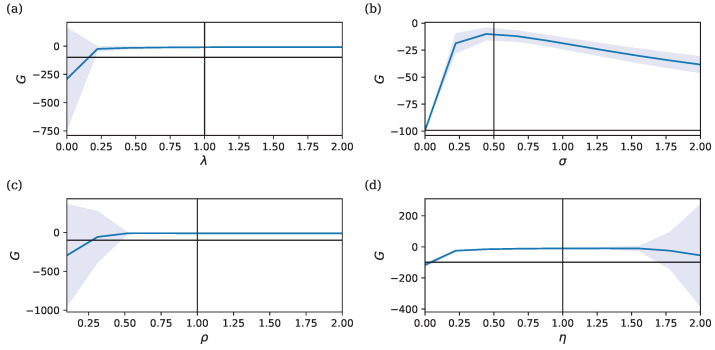
Sensitivity of the final cumulative reward G(T) to model hyper-parameters for the input–output mapping task. Results are averaged over 15 runs using different random seeds. The shaded area represents variability across runs, showing stability across a wide range of hyper-parameter settings. Vertical lines show the chosen hyper-parameter values, and horizontal lines show the return without learning. (**a**) Impact of λ. (**b**) Impact of σ. (**c**) Impact of ρ. (**d**) Impact of η.

Analyzing the sensitivity of parameter convergence to hyper-parameter choices provides valuable insights. Figure 3 shows how the choice of hyper-parameters influences the final obtained cumulative reward for the same task (input–output mapping learning). For all hyper-parameters, we see a clear peak in *G*, except for ρ since the true average reward is equal to the initial estimate of ${\bar{r}}_{0}=0$. Even for high noise levels σ, we still observe effective learning.

### 3.2. Learning a Multi-Parameter Model

Having demonstrated the feasibility of learning with a single parameter, we now investigate whether effective learning extends to cases involving multiple parameters $\theta ={\left(\theta_{1},\ldots ,\theta_{n}\right)}^{⊤}$. For this, we assume a model given by

(10) $$y=g_{\theta }\left(x\right)=\tanh\left(\theta^{⊤}x\right)$$

where $x={\left(x_{1},\ldots ,x_{n}\right)}^{⊤}$ is the input vector and *y* is the scalar output. The input x is composed of multiple sine waves, defined as $x_{i}\left(t\right)=\sin\left(i,0.1,t+\left(i-1\right)2\pi /n\right)$ for 1≤i≤n. The reward is given by $r=-{\left(y-y^{*}\right)}^{2}$ with target output $y^{*}=\tanh\left({\left(\theta^{*}\right)}^{⊤}x\right)$, generated by ground-truth parameters $\theta^{*}={\left(0.3,1.1,0.0,-0.3,-1.5,-0.4\right)}^{⊤}$. Finally, learning dynamics are given by

(11) $$\begin{array}{cc} d\theta & =\lambda (\mu -\theta )dt+d\Sigma W \end{array}$$

(12) $$\begin{array}{cc} d\mu & =\eta \delta_{r}\left(\theta -\mu \right)dt \end{array}$$

with Σ=σI and $W={\left(W_{1},\ldots ,W_{n}\right)}^{⊤}$ a multivariate standard Wiener process.

**Figure 4:**
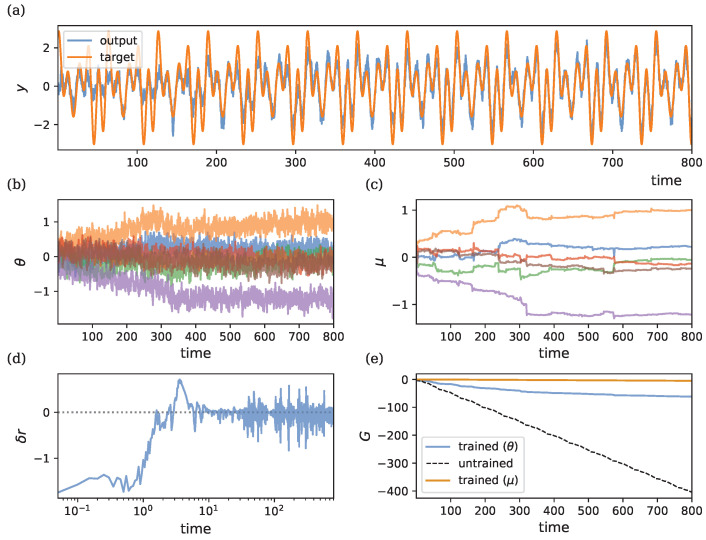
Learning dynamics in a multi-parameter model with ρ=λ=η=1 and σ=0.2. Initial conditions are ${\bar{r}}_{0}=-1$ and $\theta_{0}=\mu_{0}=0$. The ground-truth parameters used to generate the target output are $\theta^{*}={\left(0.3,1.1,0.0,-0.3,-1.5,-0.4\right)}^{⊤}$. (**a**) Target output vs. model output. (**b**) Evolution of $\theta ={\left(\theta_{1},\ldots ,\theta_{6}\right)}^{⊤}$ with individual parameter values denoted by different colors. (**c**) Evolution of $\mu ={\left(\mu_{1},\ldots ,\mu_{6}\right)}^{⊤}$ with individual mean values denoted by different colors. (**d**) RPE $\delta_{r}$, shown on a logarithmic axis to better visualize initial convergence. The dotted line denotes zero prediction error. (**e**) Cumulative reward *G*, showing improvement with learning compared to the untrained model (dashed line). The blue line indicates the cumulative reward during parameter learning. The dashed line denotes the return when θ is fixed to the initial value $\theta_{0}$. The orange line indicates the return obtained when we fix parameters to the final mean parameters θ(t)=μ(T).

Figure 4 shows that effective learning can still be achieved when having multiple parameters. Figure 4a depicts the target output vs. the model output. In Figure 4b,c, we observe the trajectories of θ and μ as they converge towards values that allow the model to approximate the target output. Figure 4d shows how the RPE $\delta_{r}$ tends to zero over time. As in the previous models, $\delta_{r}$ remains noisy after convergence due to the intrinsic stochasticity in the parameters. Figure 4e displays the cumulative reward (*G*) over time. The dashed line represents the return for an untrained model.

Note that we may also choose to use the final mean μ(T) as the parameters estimated after learning. While this diverges from the continual learning setting, it is of importance when deploying trained systems in real-world applications. The orange line in Figure 4d shows that this indeed provides optimal performance.

### 3.3. Weather Prediction Task

We now apply our approach to a real-world weather prediction task. The input vector x consists of several weather features: current temperature, humidity, wind speed, sine of the wind direction angle, cosine of the wind direction angle, and another humidity measurement. The model aims to predict the temperature, denoted as *y*, 24 h ahead. The setup follows the structure used in the multiple-parameter analysis, with the main difference being that $y^{*}$ now represents the target temperature 24 h ahead. The predicted temperature is given by $y=\theta^{⊤}x$. To validate test performance, a separate segment of the weather dataset was used. Additionally, motivated by recent work that shows that input decorrelation improves learning efficiency [51,52], we tested the model both with and without applying ZCA decorrelation [53] to the input features.

**Figure 5:**
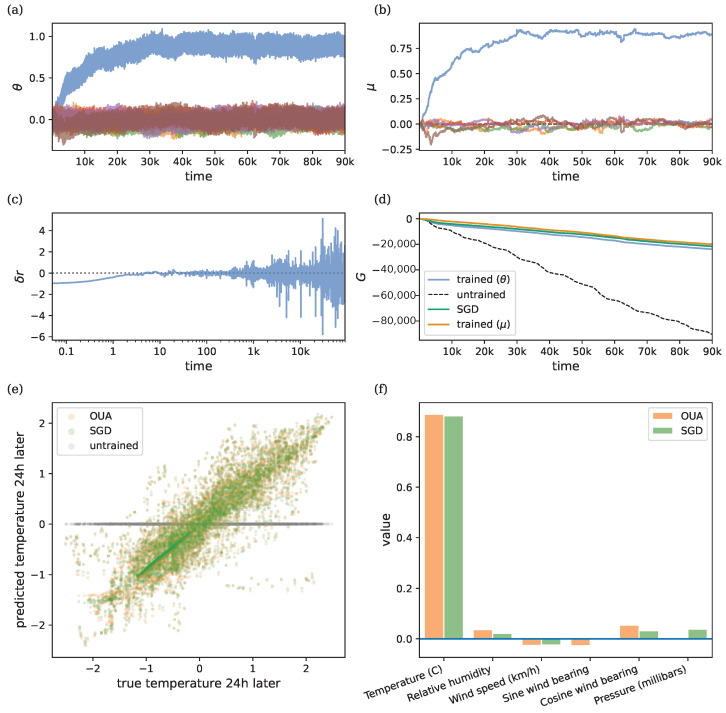
Dynamics of the weather prediction model whose parameters θ denote the different weather features. The goal is to learn to predict the temperature 24 h ahead. Hyper-parameters: ρ=λ=1, σ=0.05, and η=0.1. Initial conditions: $\bar{r}=0$, $\theta_{0}∼N\left(0,10^{-6}I\right)$ and $\mu_{0}=\theta_{0}$. (**a**) Evolution of $\theta ={\left(\theta_{1},\ldots ,\theta_{6}\right)}^{⊤}$ with individual parameter values denoted by different colors. (**b**) Evolution of $\mu ={\left(\mu_{1},\ldots ,\mu_{6}\right)}^{⊤}$ with individual mean values denoted by different colors. (**c**) RPE $\delta_{r}$, shown on a logarithmic axis to better visualize initial convergence. The dotted line denotes zero prediction error. (**d**) Cumulative reward *G* using ZCA-decorrelated data, showing improvement with learning compared to the untrained model (dashed line) and comparable performance to stochastic gradient descent (green line). The blue line indicates the return during parameter learning. The dashed line denotes the return when θ are fixed to their initial values $\theta_{0}$. The orange line indicates the return obtained when we fix parameters to the final mean parameters θ(t)=μ(T). (**e**) Predicted vs. true 24 h-ahead temperature on ZCA-decorrelated test data, using final μ(T) and initial $\theta_{0}$. (**f**) Final parameter values for each regressor using ZCA-decorrelated data.

Figure 5a–c shows the learning dynamics for this task. Figure 5d shows that, during training, the model learns to improve the cumulative reward. Figure 5e shows a scatter plot comparing the true versus predicted 24-h ahead temperature before training (gray) and after training (orange). The final mean values μ(T) were used as the parameters θ(t) when performing inference on separate test data. Figure 5f shows these final mean values, indicating that the current temperature mostly determines the prediction outcome. Figure 5d–f show results over ZCA-decorrelated data. As summarized in Table 1, the model achieves accurate predictions, with performance comparable to stochastic gradient descent (SGD), which was included as a baseline in this experiment. The OUA results were generated using the mean parameters as the model parameters, i.e., θ=μ.

### 3.4. Learning in Recurrent Systems

While the previous analysis explored learning a static input–output mapping, we now examine the learning dynamics of a non-linear recurrent system, given by

(13) $$\begin{array}{cc} dz & =(f\left(\theta_{1}z+\theta_{2}x\right)-z)dt\\ y & =\theta_{3}z \end{array}$$

where *x* is the input, *z* is the latent state, and *y* is the output. This model can be interpreted as a continuous-time recurrent neural network (CTRNN) or a latent ordinary differential equation (ODE). The reward is given by $r=-{\left(y-y^{*}\right)}^{2}$, where $y^{*}$ is the target output generated using fixed target parameters $\theta^{*}=\left(\theta_{1},\theta_{2},\theta_{3}\right)=\left(0.3,0.7,1.0\right)$. This setup requires fast dynamics in *z* and slow dynamics in θ, creating a challenging learning scenario. We demonstrate OUA’s capability to learn the parameters of a recurrent model by training it on a 1D input–output mapping.

**Figure 6:**
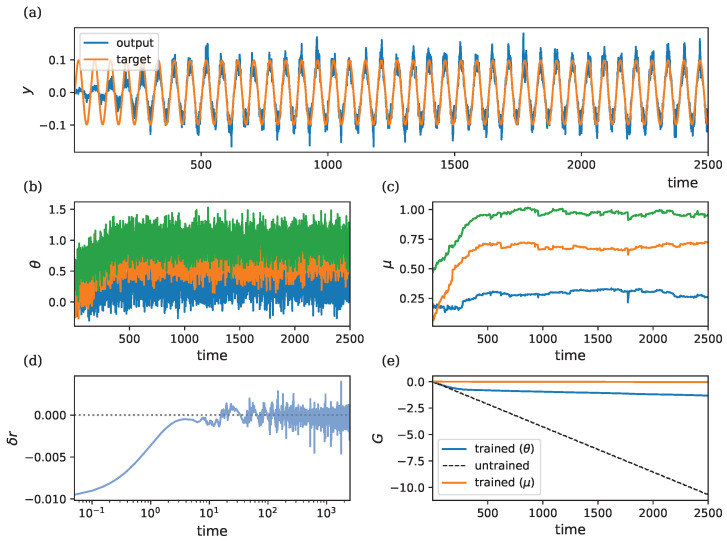
Learning dynamics in a recurrent model with ρ=λ=1, η=50, and σ=0.2. Initial conditions are ${\bar{r}}_{0}=-0.1$ and $\theta_{0}=\mu_{0}={\left(0.2,0.1,0.5\right)}^{⊤}$. The ground-truth parameters used to generate the target output are $\theta^{*}={\left(0.3,0.7,1.0\right)}^{⊤}$. (**a**) Target output vs. model output. (**b**) Evolution of $\theta ={\left(\theta_{1},\theta_{2},\theta_{3}\right)}^{⊤}$ in blue, orange and green. (**c**) Evolution of $\mu ={\left(\mu_{1},\mu_{2},\mu_{3}\right)}^{⊤}$ in blue, orange and green. (**d**) RPE $\delta_{r}$, shown on a logarithmic axis to better visualize initial convergence. The dotted line denotes zero reward prediction error. (**e**) Cumulative reward *G*, showing improvement with learning compared to the untrained model (dashed line). The blue line indicates the return during parameter learning. The dashed line denotes the return when θ are fixed to their initial values $\theta_{0}$. The orange line indicates the return obtained when we fix parameters to the final mean parameters θ(t)=μ(T).

Figure 6 illustrates the learning dynamics of this three-parameter recurrent system, which includes connections from the input to the latent state, recurrent dynamics within the latent state, and connections from the latent state to the output. Figure 6a depicts the target output vs. the model output for the different random seeds. In Figure 6b,c, we observe the trajectories of θ and μ as they converge towards values that allow the model to approximate the target output. We can observe the same probabilistic convergence behavior described in the non-recurrent model. Figure 2d shows how the RPE $\delta_{r}$ tends to zero over time. As in the non-recurrent model, $\delta_{r}$ remains noisy after convergence due to the intrinsic stochasticity in the parameters. Figure 2e displays the cumulative reward *G* over time. The dashed line represents the return for an untrained model.

### 3.5. Learning to Control a Stochastic Double Integrator

Next, we explore the task of controlling a stochastic double integrator (SDI) [48], which presents a more complex learning environment compared to the supervised learning tasks considered earlier. In this setup, the agent’s actions influence the controlled system, which in turn affects the agent’s observations. This feedback loop can lead to potentially unstable dynamics if not handled properly.

Let $s={\left(s_{1},s_{2}\right)}^{⊤}$ represent the state vector, where $s_{1}$ and $s_{2}$ denote the position and velocity of a particle moving in one dimension. The SDI system is described by the following state-space equations:

(14) $$\begin{array}{cc} ds & =\left(\left[\begin{array}{cc} 0 & 1\\ 0 & -\gamma \end{array}\right]s+\left[\begin{array}{c} 0\\ 1 \end{array}\right]y\right)t+\left[\begin{array}{c} 0\\ \alpha \end{array}\right]dW \end{array}$$

(15) $$\begin{array}{cc} x & =s+\beta \epsilon \end{array}$$

where ϵ∼N(0,I). Here, α represents process noise, β represents observation noise, and γ represents a friction term. Note that we again employ an OU process to model the stochastic dynamics of the velocity. The reward is given by a (negative) quadratic cost $r=-{0.5||s||}^{2}-0.5y^{2}$, penalizing deviations of the state s from the set-point, where both the position and velocity are equal to zero, while also penalizing large values of the control *y*. The agent is defined by $y=\theta^{⊤}x$, with learning dynamics given by Equations (8) and (9), as before. Note that the output *y* of the agent is the control input to the SDI, whereas the SDI generates the observations x to the agent. Hence, both the agent and the environment are modeled as coupled dynamical systems.

**Figure 7:**
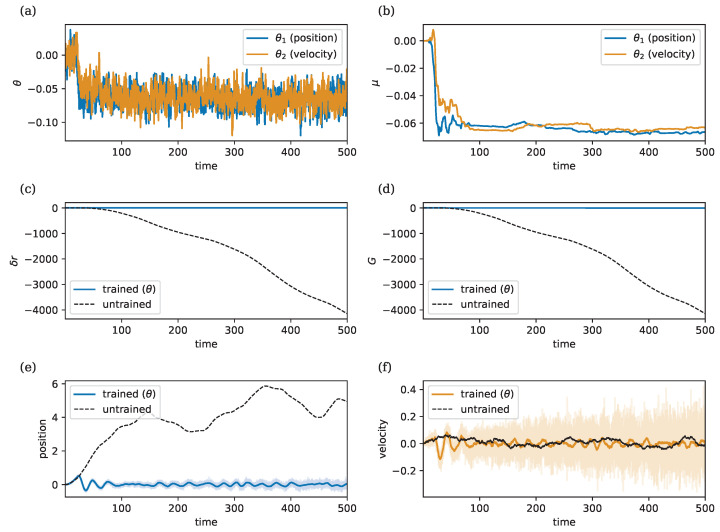
Learning to control a stochastic double integrator. Hyper-parameters: ρ=2, λ=1, σ=0.02, and η=50, γ=0.01, α=β=0.005. Initial conditions are set to zero for all variables. (**a**) Evolution of $\theta ={\left(\theta_{1},\theta_{2}\right)}^{⊤}$. (**b**) Evolution of $\mu ={\left(\mu_{1},\mu_{2}\right)}^{⊤}$. (**c**) Reward prediction error $\delta_{r}$. (**d**) Cumulative reward *G*, where higher is better. Blue line: return during learning; dashed line: return with fixed $\theta_{0}$. (**e**) Particle position after learning (blue) with observation noise (light blue); dashed line: position changes without learning. (**f**) Particle velocity after learning (orange) with observation noise (light orange); dashed line: velocity fluctuations without learning.

Figure 7 shows that OUA can learn to control a stochastic double integrator, demonstrating effective learning in this more challenging control setting. Both the parameters for the position and the velocity converge to negative values. This is indeed optimal since it induces accelerations that move the particle’s position and velocity to zero.

Figure 7 illustrates the learning dynamics of the stochastic double integrator control task. As shown in Figure 7a–c, the agent learns to adjust the parameters θ and their means μ during the learning process. Figure 7d shows that the return improves over time, with the model achieving a better performance compared to the baseline, where θ is fixed to its initial values $\theta_{0}$.

The learning process allows the agent to effectively control the particle, as seen in Figure 7e,f. The dashed lines represent the behavior of the particle without learning, showing a significant deviation of the position from zero. The learned controller, however, successfully adjusts the velocity to drive the position to zero, demonstrating that the agent has effectively learned to stabilize the system. Thus, OUA can successfully learn to control the stochastic double integrator, even in the presence of observation and process noise.

### 3.6. Meta-Learning

In the previous analyses, we estimated the parameters θ and μ while keeping the hyper-parameters fixed. However, we can also choose to learn the hyper-parameters using the same mechanism. To illustrate this, we introduce a learnable diffusion coefficient σ for the single-parameter model. Rather than fixing σ, we allow it to adjust, which in turn modulates the exploration versus exploitation trade-off in the learning process. This can be viewed as a form of meta-learning, where the adjustment of σ influences the dynamics of learning based on the problem at hand [54].

To implement meta-learning, we define additional dynamics for σ, given by

(16) $$\begin{array}{cc} d\sigma & =\lambda^{\sigma }\left(\mu^{\sigma }-\sigma \right)dt+\rho dW \end{array}$$

(17) $$\begin{array}{cc} d\mu^{\sigma }\; & =\eta^{\sigma }\delta_{r}\left(\sigma -\mu^{\sigma }\right)dt \end{array}$$

where *W* is a standard Wiener process, analogous to the equations for θ and μ in previous sections (Equations (8) and (9)).

To verify that the meta-learning process effectively identifies the optimal value for the chosen hyper-parameter, in this case, σ, we test the model in a volatile environment. For this, we tackled a simple input–output mapping task, where the target parameter $\theta^{*}$, used to generate the target output, switches from +1 to −1 during learning.

**Figure 8:**
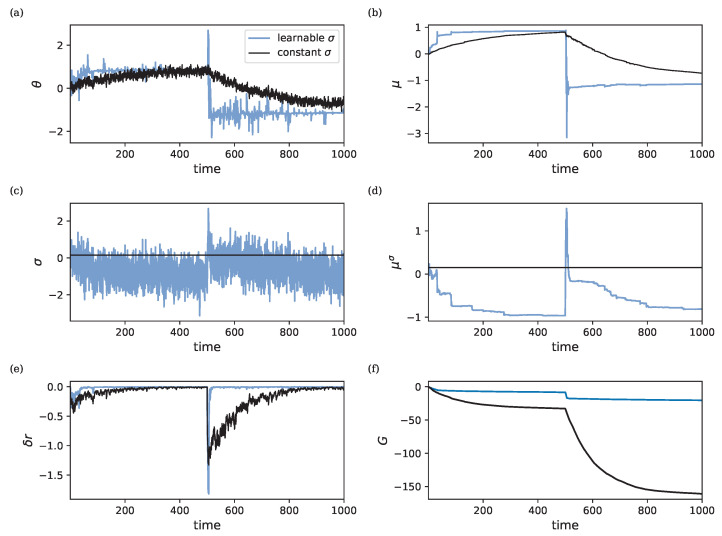
Results when using a learnable σ (blue line) compared to a constant $\sigma =\sigma_{0}$ (black line). Parameters are set to ρ=λ=η=1, $\lambda^{\sigma }=2$, and $\eta^{\sigma }=3.0$ for the model with learnable σ. The initial conditions are given by $\bar{r}=\theta_{0}=\mu_{0}=0$ and $\sigma_{0}=\mu_{0}^{\sigma }=0.15$. (**a**) Dynamics of θ. (**b**) Dynamics of μ. (**c**) Dynamics of σ. (**d**) Dynamics of $\mu^{\sigma }$. (**e**) Dynamics of $\delta_{r}$. (**f**) Dynamics of *G*.

Figure 8 presents the learning dynamics of σ under these volatile conditions. As seen in Figure 8a, the system converges faster to the optimal θ when meta-learning is employed, compared to using a fixed σ. Additionally, Figure 8 demonstrates how σ adapts by increasing its value when θ changes, promoting exploration, and decreasing when the target remains stable, favoring exploitation. The faster convergence is also reflected by the larger return in Figure 8f. Figure 8c,d show that σ and $\mu^{\sigma }$ rapidly adapt to a more suitable higher value of σ, encouraging fast exploration. At a later stage, we observe adaptation to a value of σ below the initial $\sigma_{0}$ value, encouraging exploitation.

## 4. Discussion

In this paper, we introduced Ornstein–Uhlenbeck adaptation as a learning mechanism for naturally and artificially intelligent systems. Our results show that learning can emerge purely from simulating the dynamics of parameters governed by an Ornstein–Uhlenbeck process, where mean values are updated based on a global reward prediction error. We showed that OUA effectively learns in supervised and reinforcement learning settings, including single- and multi-parameter models, recurrent systems, and meta-learning tasks. Notably, meta-learning via OUA allows the model to balance exploration and exploitation [55,56] automatically. We hypothesize that, in this setting, the remaining stochastic drift of θ around its mean approximates the posterior distribution of θ, similar to Bayesian methods [57].

OUA offers significant advantages for machine learning as it offers a gradient-free learning framework. Unlike backpropagation, no exact gradients, backward passes, or non-local information beyond access to a global reward signal are needed. OUA is also highly parallelizable since multiple agents may simultaneously explore the parameter space using one global parameter mean μ, which may have implications for federated learning [58]. Our experiments highlight the importance of hyper-parameter selection for learning stability (Figure 3), suggesting that meta-learning or black-box optimization techniques, such as Bayesian optimization [59,60] or evolution strategies [61], could further enhance OUA’s performance.

It should be noted that OUA is inherently a stochastic algorithm since noise fluctuations drive learning. This means that, in the limit, instead of converging towards exact parameter values, individual parameter values θ fluctuate about their mean μ with variance $\sigma^{2}2\lambda$. This is also reflected by induced RPE fluctuations around zero. We consider this behavior a feature rather than a bug since it allows the agent to adapt to changing circumstances by continuously probing for better parameter values. This can be seen in Figure 8, where the system responds to volatile target parameters. As also shown in this figure, the meta-learning formulation further allows the system to reduce parameter variance in stable situations. Suppose one does want to enforce fixed parameter values, as shown in Figure 4 and Figure 5, one may choose to set the parameters θ to their estimated mean values μ, or enforce convergence by increasing the rate parameter λ (or decreasing the noise variance $\sigma^{2}$) over time, similar to the use of learning rate schedulers in conventional neural network training.

A key area for future work is scaling OUA to more complex tasks. This could involve replacing Wiener processes with other noise processes, such as compound Poisson processes, to selectively update subsets of parameters, potentially reducing weight entanglement. Additionally, as demonstrated in Figure 5, decorrelating the input data via ZCA whitening accelerates convergence [51,52]. Such decorrelation strategies can generalize across deep and recurrent networks [33,34] and even be implemented locally as part of the forward dynamics [52]. OUA is also well-suited for training deep networks, which can be interpreted in our framework as recurrent networks with block-diagonal structures.

OUA might be relevant for biological learning as well since it suggests potential mechanisms for noise-based learning in the brain [62,63]. Here, we draw a connection with the stochastic nature of neurotransmitter release [64,65], which introduces variability in synaptic transmission, akin to the exploratory noise in OUA’s parameter updates. Hypothetically, this stochasticity, paired with global reward signals such as dopaminergic RP [44,66], could guide synaptic weight adjustments dynamically, as modeled by the mean-reverting updates in OUA. Whether or not such noise-based mechanisms are at play in biological learning remains an open question and requires experimental validation. OUA also shares conceptual similarities with reward-modulated Hebbian learning, where the update of the synaptic weight $\theta_{ij}$ is a function of the pre- and post-synaptic activity (specifically, the activity deviation from their mean activity) and the RPEs [40]. Formally, while RMHL provides a biologically plausible interpretation of node perturbation [28,30,31,32,33,34], OUA offers a biologically plausible interpretation of weight perturbation [37,38,39]. It is important to note that, while our framework is inspired by biological processes, it operates at a conceptual level rather than by simulating specific neurophysiological mechanisms. Importantly, the conceptual parallels we draw to brain processes serve to provide a high-level understanding of how noise and local reward signals might facilitate efficient learning in biological systems. That is, we do not aim to model detailed neurophysiological processes but to leverage these principles in a computational framework that remains biologically inspired.

Our work is of particular relevance for neuromorphic computing and other unconventional (non-von-Neumann-style) architectures [19,67,68,69,70,71,72], where learning and inference emerge from the physical dynamics of the system [15,73]. These approaches pave the way for sustainable, energy-efficient intelligent systems. Unlike conventional AI, where learning and inference are treated as distinct processes, our method integrates them seamlessly. It relies solely on the online adaptation of parameter values through drift and diffusion terms, which can be directly implemented in physical systems. OUA’s reliance on local, continuous-time parameter updates makes it a natural fit for spiking neuromorphic systems [74], eliminating the need for differentiability or separate forward and backward stages for inference and learning.

Looking ahead, we envision future AI systems where intelligence emerges purely from running a system’s equations of motion forward in time to maximize efficiency and effectiveness. OUA exemplifies how such physical learning machines could be realized through local operations, leveraging the mean-reverting dynamics of the Ornstein–Uhlenbeck process. Future work will focus on theoretical extensions, scaling OUA to high-dimensional tasks, addressing challenges like delayed rewards and catastrophic forgetting, and implementing OUA on neuromorphic and unconventional computing platforms.

## Figures and Tables

**Table 1:** Test performance comparison between SGD and OUA on the weather task. Included metrics are MSE and Pearson correlation using both ZCA-decorrelated data and original data.

	ZCA Data		Original Data	
	**MSE**	**Pearson Corr.**	**MSE**	**Pearson Corr.**
SGD	0.21	0.871	0.21	0.874
OUA	0.22	0.871	0.22	0.871

## Data Availability

The weather data used in this study are openly available in Kaggle at https://www.kaggle.com/datasets/budincsevity/szeged-weather/ (accessed on 20 December 2024).
